# Synthesis and crystal structure of 1*H*-1,2,4-triazole-3,5-di­amine monohydrate

**DOI:** 10.1107/S2056989024009265

**Published:** 2024-10-11

**Authors:** Kazuki Inoue, Shun Nakami, Mieko Kumasaki, Shinya Matsumoto

**Affiliations:** a Graduate School of Environment and Information Sciences Yokohama National University, 79-7 Tokiwadai Hodogaya-ku Yokohama Kanagawa 240-8501 Japan; b Faculty of Environment and Information Sciences Yokohama National University, 79-7 Tokiwadai Hodogaya-ku Yokohama-shi Kanagawa 240-8501 Japan; Indian Institute of Science Education and Research Bhopal, India

**Keywords:** guanazole, 3,5-di­amino-1,2,4-triazole, hydrate, sodium perchlorate, crystal structure

## Abstract

The title compound, a hydrate of 3,5-di­amino-1,2,4-triazole (DATA), C_2_H_5_N_5_·H_2_O, was synthesized in the presence of sodium perchlorate. The evaporation of H_2_O from its aqueous solution resulted in anhydrous DATA, suggesting that sodium perchlorate was required to precipitate the DATA hydrate. The DATA hydrate crystallizes in the *P*2_1_/*c* space group in the form of needle-shaped crystals with one DATA and one water mol­ecule in the asymmetric unit. The water mol­ecules form a three-dimensional network in the crystal structure. Hirshfeld surface analysis revealed that 8.5% of the inter­molecular inter­actions originate from H⋯O contacts derived from the incorporation of the water mol­ecules.

## Abstract 2

1*H*-1,2,4-Triazole-3,5-di­amine monohydrate was synthesized and its crystal structure was determined. Two 3,5-di­amino-1,2,4-triazole mol­ecules form mutual hydrogen bonds.

## 1. Chemical context

Researchers have focused on the development of less sensitive and highly energetic materials. The sensitivity of energetic materials is related to their crystal structures and inter­molecular inter­actions (Kuklja & Rashkeev, 2007 ▸). Additionally, the density, which strongly influences the detonation performance, can be calculated from the crystal structure. Hence, the determination of the crystal structure can elucidate the characteristics of energetic materials.

Azole derivatives have been recognized as promising frameworks for energetic materials because of their high heats of formation (Fisher *et al.*, 2012 ▸; Kumasaki *et al.*, 2021 ▸; Inoue *et al.*, 2022*a* ▸). Tetra­zoles, triazoles, and imidazoles are N-rich heterocyclic azole derivatives. Several energetic materials, including organic explosives, energetic salts, and co-crystals, have been synthesized using azole compounds (Kumasaki *et al.*, 2011 ▸; Mori *et al.*, 2021 ▸; Inoue *et al.*, 2022*b* ▸).
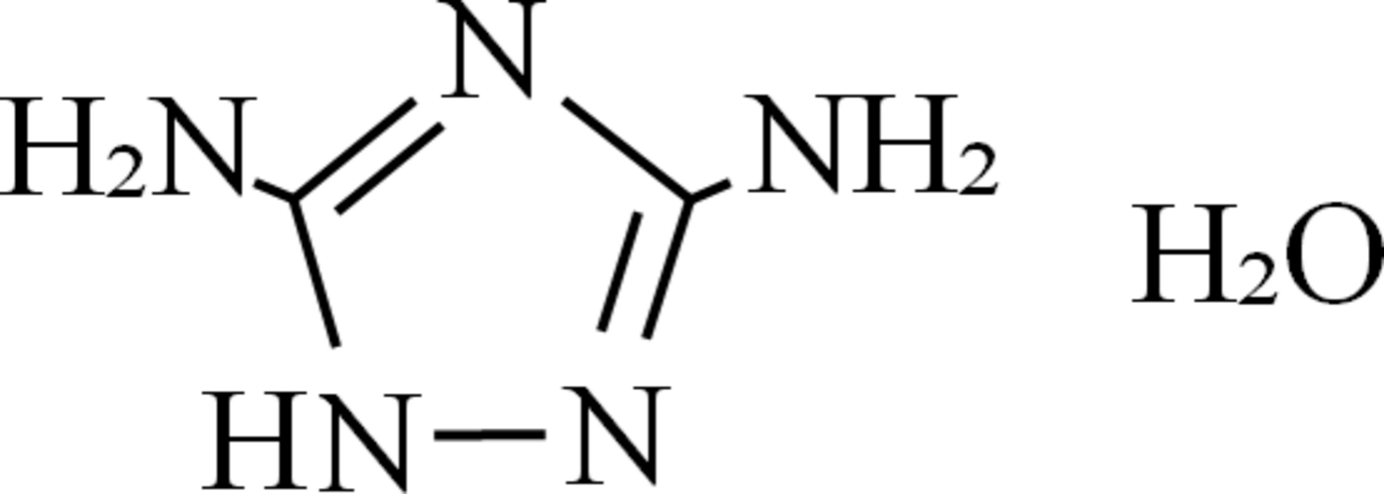


In our previous study, 1*H*-tetra­zole was co-crystallized with NaClO_4_; NaClO_4_ is an oxidizer that is sometimes used in pyrotechnics (Inoue *et al.*, 2022*b* ▸). The co-crystal exhibited high sensitivity, which was comparable to that of typical primary explosives (Inoue *et al.*, 2022*a* ▸). Subsequently, 3,5-di­amino-1,2,4-triazole (guanazole, DATA) was selected as the target material for co-crystallization with NaClO_4_. However, our attempt to prepare a co-crystal of DATA and NaClO_4_ resulted instead in crystals of DATA hydrate, which has not been previously been reported. Although DATA is used as a raw material in the synthesis of various energetic compounds (Khan *et al.*, 2024 ▸; Zhang *et al.*, 2010 ▸; Yin *et al.*, 2015 ▸), understanding its hydration is valuable for its treatment.

## 2. Structural commentary

**Figure 1:**
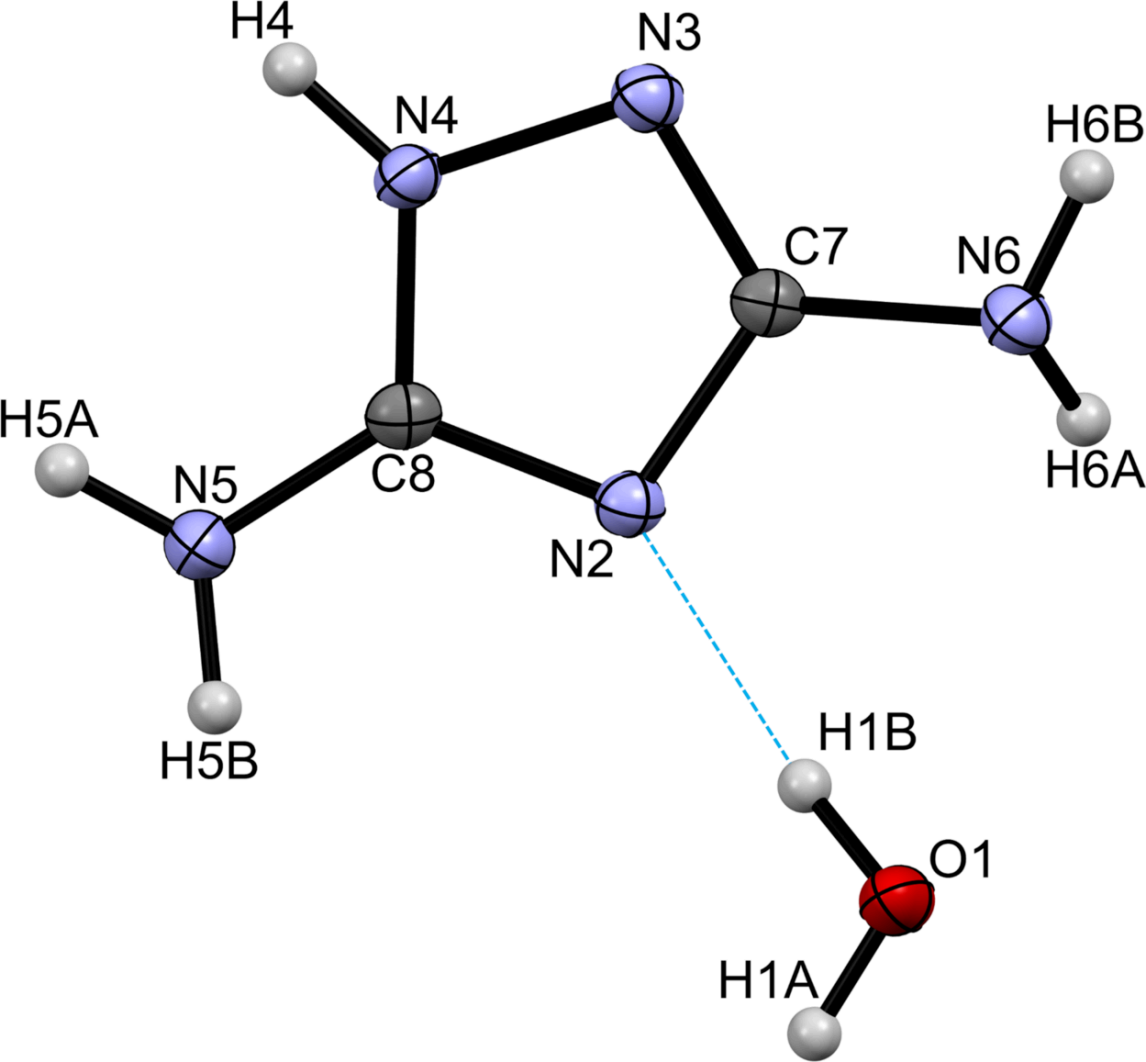
Displacement ellipsoid plot (probability level of 50%) of DATA hydrate showing the atom-numbering scheme. The hydrogen bond is represented by a dashed blue line.

DATA hydrate (Fig. 1 ▸) crystallizes in the *P*2_1_/*c* space group with one DATA mol­ecule and one water mol­ecule in the asymmetric unit. The two N atoms of the amino groups are not coplanar with the mean plane of the ring structure, the distances between the mean plane of the ring structure and N5 and N6 being 0.0719 (19) and 0.1038 (19) Å, respectively. The N4—N3 bond [1.3943 (13) Å] is longer than all of the C—N bonds [the range is 1.3797 (15) for C7—N6 to 1.3185 (15) Å for N3—C7]. Furthermore, the N2—C8 double bond [1.3400 (15) Å] is longer than C8—N4 [1.3337 (15) Å], which is a single bond. Table 1 ▸ compares the bond lengths of hydrated and non-hydrated DATA (Klapötke *et al.*, 2010 ▸). Evidently, the N2—C8 and N2—C7 bonds in DATA hydrate are longer than those of non-hydrated DATA. The differences between all of the bond lengths are statistically significant.

## 3. Supra­molecular features

**Figure 2:**
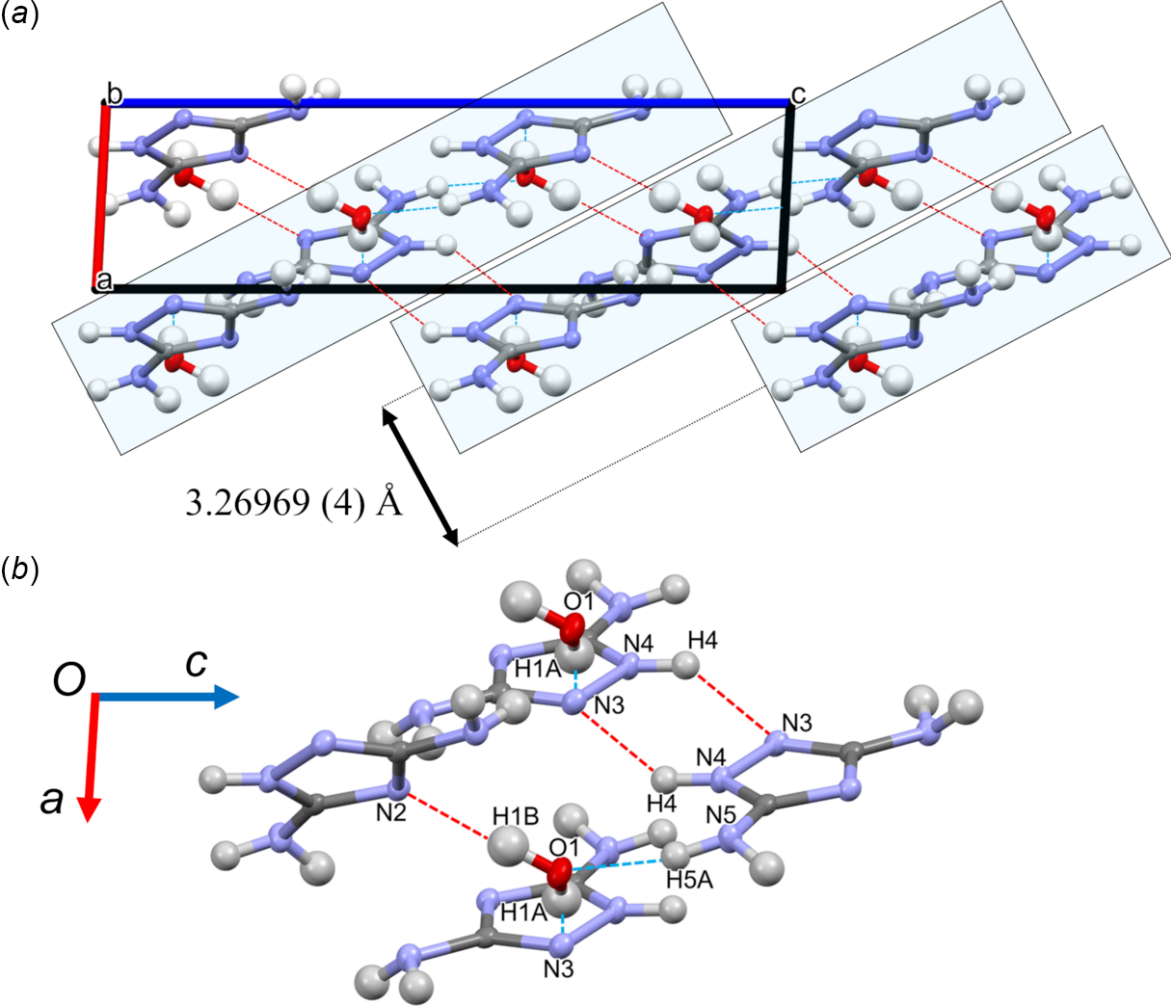
(*a*) The crystal structure viewed along the *b* axis. The intra- and inter­layer hydrogen bonds are indicated by blue and red lines, respectively, and the layer structure is shown in blue. (*b*) The intra- and inter­layer hydrogen bonds with atom numbers.

**Figure 3:**
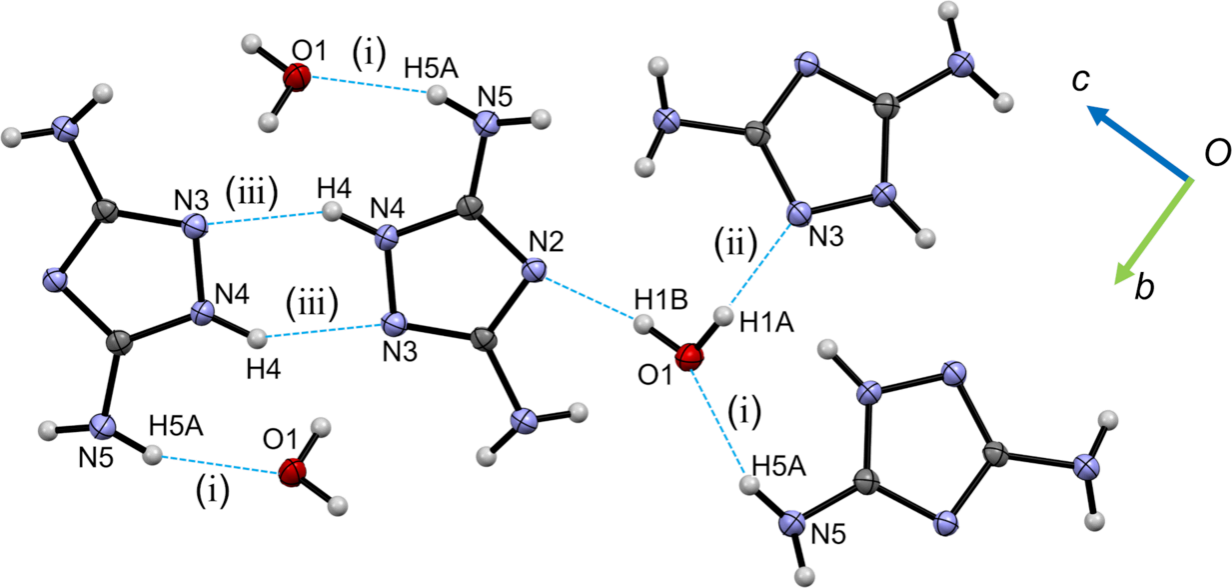
Hydrogen bonding in DATA hydrate viewed along the *a* axis. Symmetry codes: (i) *x*, 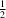 − *y*, 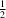 + *z*; (ii) 1 − *x*, 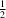 + *y*, 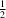 − *z*; (iii) −*x*, −*y*, −*z*.

In the crystal, the DATA and H_2_O mol­ecules form a layered structure (Fig. 2 ▸), with layers parallel to the (102) plane and an inter­layer distance of 3.26969 (4) Å. The O1—H1*A*⋯N3 and O1⋯H5*A*—N5 hydrogen bonds form the layers while the O1—H1*B*⋯N2 and N4—H4⋯N3 hydrogen bonds connect adjacent layers (Table 2 ▸, Fig. 3 ▸). The water mol­ecules produce a 3D network within the crystal. A water mol­ecule forms two hydrogen bonds with two DATA mol­ecules from the same layer [O1—H1*A*⋯N3(1 − *x*, 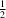 + *y*, 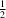 − *z*) and O1⋯H5*A*—N5(*x*, 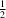 − *y*, 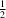 + *z*)] and one with that from an adjacent layer (O1—H1*B*⋯N2). Atoms N5 of the amino group and N2 from the ring form hydrogen bonds with each water mol­ecule; however, N6 is not involved in hydrogen bonding. Two DATA mol­ecules in two adjacent layers are mutually connected by two N4—H4⋯N3(−*x*, −*y*, −*z*) hydrogen bonds.

**Figure 4:**
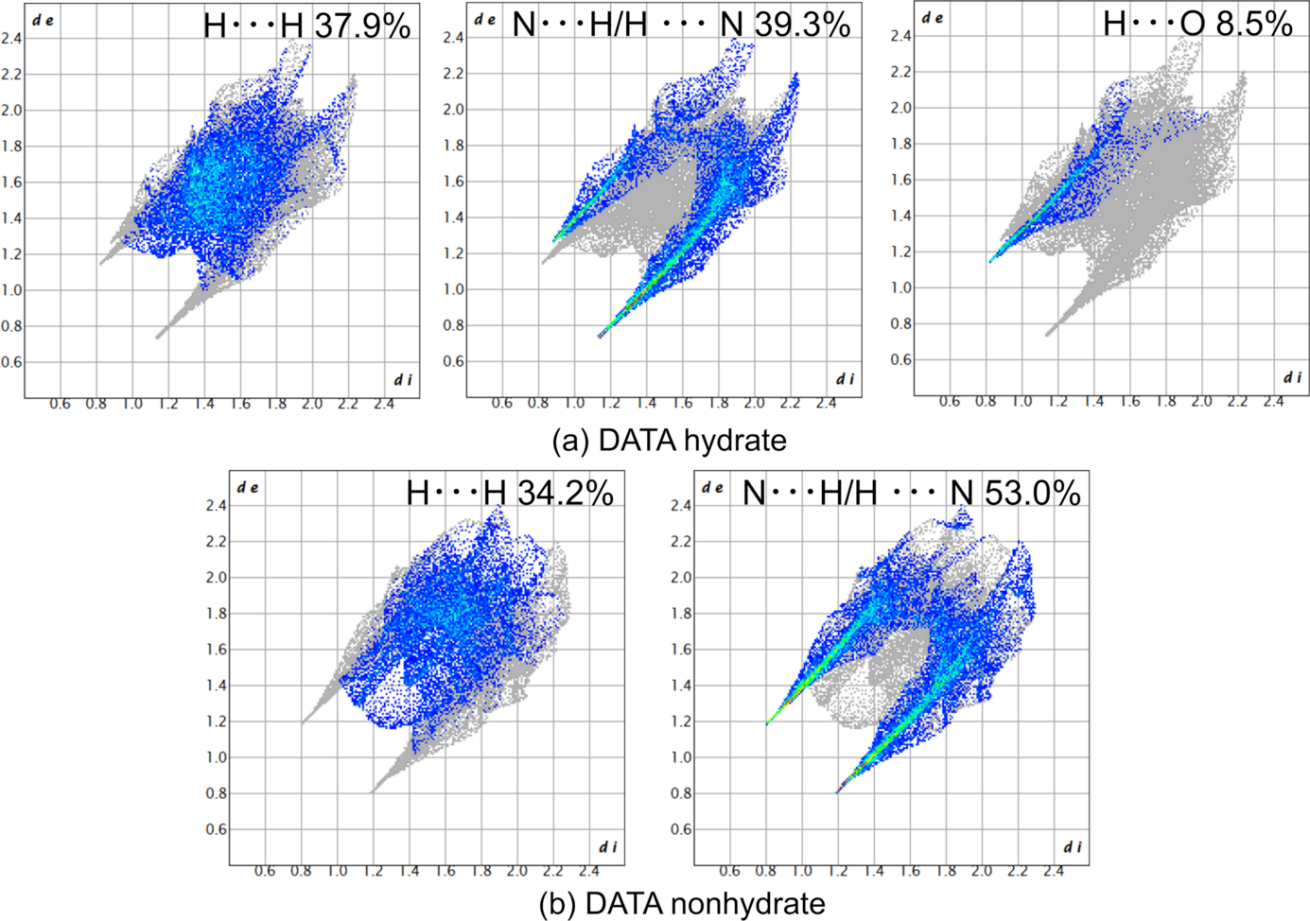
Fingerprint plots for (*a*) DATA hydrate and (*b*) anhydrous DATA.

The supra­molecular inter­actions in DATA hydrate were further investigated through Hirshfeld surface analysis using *Crystal Explorer 21* (Spackman *et al.*, 2021 ▸). Fig. 4 ▸ shows the fingerprint plots for a mol­ecule of DATA non-hydrate (Klapötke *et al.*, 2010 ▸) and DATA hydrate. The third spike concerning the H⋯O inter­action was observed upon hydration, whereas the DATA non-hydrate exhibited two spikes of N⋯H and H⋯N. The dominant inter­action of DATA non-hydrate was N⋯H/H⋯N of 53.0%, which decreased to 39.3% with the incorporation of water mol­ecules, and the H⋯O inter­action contributes 8.5% to the crystal packing. The contribution of H⋯H inter­actions in DATA hydrate is 37.9%, which is higher than that of DATA non-hydrate (34.2%).

## 4. Database survey

Previous studies on DATA were explored in the Cambridge Structural Database (CSD, June 2024; Groom *et al.*, 2016 ▸). The search resulted in four reports: DAMTRZ11 (Klapötke *et al.*, 2010 ▸), DAMTRZ22 (Ivanova & Spiteller, 2017 ▸), DAMTRZ10 (Starova *et al.*, 1979 ▸) and DAMTRZ20 (Starova *et al.*, 1980 ▸). The title DATA hydrate forms a layered structure in a monoclinic space group, whereas anhydrous DATA was reported to form a herringbone structure.

## 5. Synthesis and crystallization

DATA was purchased from Tokyo Chemical Industry Co., Ltd. Sodium perchlorate was obtained from Kanto Chemical Co., Inc. DATA (1 mmol) and sodium perchlorate (1 mmol) were dissolved in deionized water, and the solvent was removed in a silica gel desiccator. After one week, needle-shaped crystals were precipitated (yield: 40.81%). Inter­estingly, the evaporation of water from an aqueous solution of DATA generated block-shaped non-hydrated DATA crystals. Therefore, sodium perchlorate was required to precipitate the DATA hydrate. The mass proportion of H_2_O in the crystals was measured using thermogravimetry. A Thermoplus TG8120 (Rigaku) was used with an Al_2_O_3_ open cell. The heating rate was set to 10 K min^−1^. Flow gas was not used to prevent dehydration under the dried flow gas. The measured mass proportion of H_2_O in the crystal was 14.51%, which was slightly lower than the theoretical mass content of H_2_O (15.38%).

## 6. Dehydration behavior

After the storage of DATA hydrate over one night at 33% RH (saturated salt method (Greenspan, 1977 ▸); MgCl, 295 K), the water in the hydrate was removed and DATA hydrate turned into DATA. In contrast, after the storage at room temperature (approximately 295 K) and 60% RH for one night, the crystals remained as hydrates.

## 7. Refinement

The crystal data, data collection, and structural refinement details are summarized in Table 3 ▸. The H atoms were identified using difference-Fourier maps and all H-atom parameters were refined.

## Supplementary Material

Crystal structure: contains datablock(s) I. DOI: 10.1107/S2056989024009265/dx2060sup1.cif

Structure factors: contains datablock(s) I. DOI: 10.1107/S2056989024009265/dx2060Isup2.hkl

Supporting information file. DOI: 10.1107/S2056989024009265/dx2060Isup3.cml

CCDC reference: 2251960

Additional supporting information:  crystallographic information; 3D view; checkCIF report

## Figures and Tables

**Table 1:** Bond lengths (Å) in hydrated and non-hydrated DATA

Bond	DATA	DATA hydrate
C7—N2	1.3544 (16)	1.3670 (15)
N2—C8	1.3339 (16)	1.3400 (15)
C8—N4	1.3356 (16)	1.3337 (15)
N4—N3	1.3951 (15)	1.3943 (13)
N3—C7	1.3238 (16)	1.3185 (15)
C7—N6	1.3747 (17)	1.3797 (15)
C8—N5	1.3502 (17)	1.3547 (15)

**Table 2:** Hydrogen-bond geometry (Å, °)

*D*—H⋯*A*	*D*—H	H⋯*A*	*D*⋯*A*	*D*—H⋯*A*
N5—H5*A*⋯O1^i^	0.895 (17)	2.080 (17)	2.9173 (14)	155.4 (14)
O1—H1*A*⋯N3^ii^	0.871 (19)	1.988 (19)	2.8558 (13)	174.1 (16)
O1—H1*B*⋯N2	0.90 (2)	1.95 (2)	2.8410 (13)	171.5 (18)
N4—H4⋯N3^iii^	0.883 (17)	2.306 (16)	2.9947 (14)	134.9 (13)

Symmetry codes: (i) 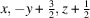; (ii) 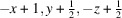; (iii) 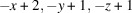.

**Table 3:** Experimental details

Crystal data	
Chemical formula	C_2_H_5_N_5_·H_2_O
*M*_r_	117.13
Crystal system, space group	Monoclinic, *P*2_1_/*c*
Temperature (K)	123
*a*, *b*, *c* (Å)	3.80560 (5), 9.49424 (11), 14.01599 (15)
β (°)	92.9639 (11)
*V* (Å^3^)	505.74 (1)
*Z*	4
Radiation type	Cu *K*α
μ (mm^−1^)	1.07
Crystal size (mm)	0.2 × 0.1 × 0.1

Data collection	
Diffractometer	XtaLAB AFC12 (RINC): Kappa dual home/near
Absorption correction	Multi-scan (*CrysAlis PRO*; Rigaku OD, 2019 ▸)
*T*_min_, *T*_max_	0.883, 1.000
No. of measured, independent and observed [*I* > 2σ(*I*)] reflections	2894, 989, 956
*R*_int_	0.019
(sin θ/λ)_max_ (Å^−1^)	0.619

Refinement	
*R*[*F*^2^ > 2σ(*F*^2^)], *wR*(*F*^2^), *S*	0.031, 0.081, 1.11
No. of reflections	989
No. of parameters	102
H-atom treatment	All H-atom parameters refined
Δρ_max_, Δρ_min_ (e Å^−3^)	0.19, −0.22

Computer programs: *CrysAlis PRO* (Rigaku OD, 2019 ▸), *SHELXT2018/2* (Sheldrick, 2015*a* ▸), *SHELXL2019/3* (Sheldrick, 2015*b* ▸), *Mercury* (Macrae *et al.*, 2020 ▸), *publCIF* (Westrip, 2010 ▸) and *OLEX2* (Dolomanov *et al.*, 2009 ▸).

## supplementary crystallographic information

### 1H-1,2,4-Triazole-3,5-diamine monohydrate Crystal data

**Table:**

C_2_H_5_N_5_·H_2_O	*F*(000) = 248
*M**_r_* = 117.13	*D*_x_ = 1.538 Mg m^−^^3^
Monoclinic, *P*2_1_/*c*	Cu *K*α radiation, λ = 1.54184 Å
*a* = 3.80560 (5) Å	Cell parameters from 2506 reflections
*b* = 9.49424 (11) Å	θ = 6.3–72.5°
*c* = 14.01599 (15) Å	µ = 1.07 mm^−^^1^
β = 92.9639 (11)°	*T* = 123 K
*V* = 505.74 (1) Å^3^	Block, clear light colourless
*Z* = 4	0.2 × 0.1 × 0.1 mm

### 1H-1,2,4-Triazole-3,5-diamine monohydrate Data collection

**Table:**

XtaLAB AFC12 (RINC): Kappa dual home/near diffractometer	989 independent reflections
Radiation source: micro-focus sealed X-ray tube, Rigaku (Cu) X-ray Source	956 reflections with *I* > 2σ(*I*)
Mirror monochromator	*R*_int_ = 0.019
Detector resolution: 5.8140 pixels mm^-1^	θ_max_ = 72.7°, θ_min_ = 5.6°
ω scans	*h* = −4→4
Absorption correction: multi-scan (CrysAlisPro; Rigaku OD, 2019)	*k* = −6→11
*T*_min_ = 0.883, *T*_max_ = 1.000	*l* = −17→16
2894 measured reflections	

### 1H-1,2,4-Triazole-3,5-diamine monohydrate Refinement

**Table:**

Refinement on *F*^2^	Hydrogen site location: difference Fourier map
Least-squares matrix: full	All H-atom parameters refined
*R*[*F*^2^ > 2σ(*F*^2^)] = 0.031	*w* = 1/[σ^2^(*F*_o_^2^) + (0.0403*P*)^2^ + 0.1848*P*] where *P* = (*F*_o_^2^ + 2*F*_c_^2^)/3
*wR*(*F*^2^) = 0.081	(Δ/σ)_max_ < 0.001
*S* = 1.11	Δρ_max_ = 0.19 e Å^−^^3^
989 reflections	Δρ_min_ = −0.22 e Å^−^^3^
102 parameters	Extinction correction: *SHELXL2019/2* (Sheldrick, 2015b), Fc^*^=kFc[1+0.001xFc^2^λ^3^/sin(2θ)]^-1/4^
0 restraints	Extinction coefficient: 0.0108 (15)
Primary atom site location: dual	

### 1H-1,2,4-Triazole-3,5-diamine monohydrate Special details

**Table:**

Geometry. All esds (except the esd in the dihedral angle between two l.s. planes) are estimated using the full covariance matrix. The cell esds are taken into account individually in the estimation of esds in distances, angles and torsion angles; correlations between esds in cell parameters are only used when they are defined by crystal symmetry. An approximate (isotropic) treatment of cell esds is used for estimating esds involving l.s. planes.

### 1H-1,2,4-Triazole-3,5-diamine monohydrate Fractional atomic coordinates and isotropic or equivalent isotropic displacement parameters (Å^2^)

**Table:**

	*x*	*y*	*z*	*U*_iso_*/*U*_eq_	
O1	0.3849 (2)	0.75354 (9)	0.11841 (6)	0.0209 (3)	
N2	0.7285 (2)	0.72150 (10)	0.30194 (7)	0.0148 (3)	
N4	0.7684 (2)	0.62922 (10)	0.44551 (7)	0.0157 (3)	
N3	0.9151 (3)	0.52932 (10)	0.38648 (7)	0.0164 (3)	
N6	0.9638 (3)	0.52599 (11)	0.21830 (7)	0.0172 (3)	
N5	0.5282 (3)	0.86004 (11)	0.43097 (8)	0.0186 (3)	
C8	0.6633 (3)	0.74110 (12)	0.39403 (8)	0.0145 (3)	
C7	0.8788 (3)	0.59072 (12)	0.30217 (8)	0.0141 (3)	
H4	0.765 (4)	0.6124 (16)	0.5074 (12)	0.021 (4)*	
H5A	0.454 (4)	0.8506 (17)	0.4902 (12)	0.023 (4)*	
H6A	1.064 (4)	0.5847 (19)	0.1783 (12)	0.032 (4)*	
H6B	1.085 (4)	0.448 (2)	0.2284 (12)	0.031 (4)*	
H5B	0.402 (4)	0.9142 (19)	0.3880 (12)	0.032 (4)*	
H1A	0.280 (5)	0.835 (2)	0.1149 (13)	0.041 (5)*	
H1B	0.495 (5)	0.753 (2)	0.1769 (16)	0.045 (5)*	

### 1H-1,2,4-Triazole-3,5-diamine monohydrate Atomic displacement parameters (Å^2^)

**Table:**

	*U*^11^	*U*^22^	*U*^33^	*U*^12^	*U*^13^	*U*^23^
O1	0.0267 (5)	0.0192 (5)	0.0162 (5)	0.0054 (4)	−0.0036 (4)	−0.0011 (3)
N2	0.0157 (5)	0.0133 (5)	0.0150 (5)	0.0000 (4)	−0.0010 (4)	0.0002 (4)
N4	0.0199 (5)	0.0147 (5)	0.0123 (5)	0.0009 (4)	−0.0014 (4)	0.0003 (4)
N3	0.0181 (5)	0.0142 (5)	0.0165 (5)	0.0011 (4)	−0.0018 (4)	−0.0009 (4)
N6	0.0211 (5)	0.0136 (5)	0.0169 (5)	0.0012 (4)	0.0003 (4)	−0.0005 (4)
N5	0.0239 (5)	0.0164 (5)	0.0156 (5)	0.0037 (4)	0.0011 (4)	0.0001 (4)
C8	0.0130 (5)	0.0144 (6)	0.0157 (5)	−0.0021 (4)	−0.0022 (4)	0.0002 (4)
C7	0.0128 (5)	0.0128 (5)	0.0165 (6)	−0.0018 (4)	−0.0018 (4)	−0.0001 (4)

### 1H-1,2,4-Triazole-3,5-diamine monohydrate Geometric parameters (Å, º)

**Table:**

O1—H1A	0.87 (2)	N3—C7	1.3185 (15)
O1—H1B	0.90 (2)	N6—C7	1.3797 (15)
N2—C8	1.3400 (15)	N6—H6A	0.891 (19)
N2—C7	1.3670 (15)	N6—H6B	0.884 (19)
N4—N3	1.3943 (13)	N5—C8	1.3547 (15)
N4—C8	1.3337 (15)	N5—H5A	0.896 (17)
N4—H4	0.883 (16)	N5—H5B	0.910 (18)
			
H1A—O1—H1B	104.3 (17)	C8—N5—H5A	114.5 (10)
C8—N2—C7	102.83 (9)	C8—N5—H5B	114.6 (10)
N3—N4—H4	119.2 (10)	H5A—N5—H5B	119.2 (14)
C8—N4—N3	109.80 (9)	N2—C8—N5	125.22 (10)
C8—N4—H4	131.0 (10)	N4—C8—N2	110.20 (10)
C7—N3—N4	101.79 (9)	N4—C8—N5	124.49 (11)
C7—N6—H6A	112.7 (11)	N2—C7—N6	121.27 (10)
C7—N6—H6B	112.5 (11)	N3—C7—N2	115.36 (10)
H6A—N6—H6B	112.9 (15)	N3—C7—N6	123.25 (11)
			
N4—N3—C7—N2	1.14 (12)	C8—N2—C7—N6	175.30 (10)
N4—N3—C7—N6	−174.87 (10)	C8—N4—N3—C7	−1.05 (12)
N3—N4—C8—N2	0.66 (13)	C7—N2—C8—N4	0.04 (12)
N3—N4—C8—N5	−176.03 (10)	C7—N2—C8—N5	176.69 (11)
C8—N2—C7—N3	−0.79 (12)		

### 1H-1,2,4-Triazole-3,5-diamine monohydrate Hydrogen-bond geometry (Å, º)

**Table:**

*D*—H···*A*	*D*—H	H···*A*	*D*···*A*	*D*—H···*A*
N5—H5*A*···O1^i^	0.895 (17)	2.080 (17)	2.9173 (14)	155.4 (14)
O1—H1*A*···N3^ii^	0.871 (19)	1.988 (19)	2.8558 (13)	174.1 (16)
O1—H1*B*···N2	0.90 (2)	1.95 (2)	2.8410 (13)	171.5 (18)
N4—H4···N3^iii^	0.883 (17)	2.306 (16)	2.9947 (14)	134.9 (13)

Symmetry codes: (i) *x*, −*y*+3/2, *z*+1/2; (ii) −*x*+1, *y*+1/2, −*z*+1/2; (iii) −*x*+2, −*y*+1, −*z*+1.
